# Red Fluorescent Genetically Encoded Voltage Indicators with Millisecond Responsiveness

**DOI:** 10.3390/s19132982

**Published:** 2019-07-06

**Authors:** Liubov A. Kost, Violetta O. Ivanova, Pavel M. Balaban, Konstantin A. Lukyanov, Evgeny S. Nikitin, Alexey M. Bogdanov

**Affiliations:** 1 Shemyakin-Ovchinnikov Institute of Bioorganic Chemistry, Moscow 117997, Russia; 2 Institute of Higher Nervous Activity and Neurophysiology, Moscow 117485, Russia; 3 Center of Life Sciences, Skolkovo Institute of Science and Technology, Moscow 121205, Russia

**Keywords:** biosensing techniques, electrophysiology, membrane voltage, voltage sensors, genetically encoded voltage indicators, interdomain linkers, red fluorescent proteins

## Abstract

Genetically encoded fluorescent indicators typically consist of the sensitive and reporter protein domains connected with the amino acid linkers. The final performance of a particular indicator may depend on the linker length and composition as strong as it depends on the both domains nature. Here we aimed to optimize interdomain linkers in VSD-FR189-188—a recently described red fluorescent protein-based voltage indicator. We have tested 13 shortened linker versions and monitored the dynamic range, response speed and polarity of the corresponding voltage indicator variants. While the new indicators didn’t show a contrast enhancement, some of them carrying very short interdomain linkers responded 25-fold faster than the parental VSD-FR189-188. Also we found the critical linker length at which fluorescence response to voltage shift changes its polarity from negative to positive slope. Our observations thus make an important contribution to the designing principles of the fluorescent protein-derived voltage indicators.

## 1. Introduction

Fluorescent proteins (FP) constitute a basis for a diverse class of molecular tools called FP-based molecular sensors or FP-based genetically encoded indicators. In molecular sensors, changes of FP fluorescence are used to visualize diverse physicochemical parameters *in vitro*, in living cells and even at the whole-organism level. Currently there are dozens of sensors designed to measure changes of concentrations of Ca^2+^ and other cations (Mg^2+^, Mo^2+^, Cu^2+^, Zn^2+^), Cl^−^, H_2_O_2_, organic metabolites (cAMP/cGMP, ATP, sugars, amino acids, etc.), to monitor cellular redox status, pH, mechanical forces, enzyme activity and membrane voltage. Basically, any genetically encoded fluorescent indicator consists of two functional parts. One of those parts is sensitive and specifically interacts with the analyte. A reporter provides signal output for the second part. Depending on the particular sensor architecture these functional parts can be represented by a single FP or several structural domains. For the multidomain sensors, the structural domains nature, general topology of their polypeptide chains, and the details of how domains are connected determine practical characteristics [1,2].

Circular permutation, domains insertion and other genetic engineering manipulations are widely used in sensor designing. At least six polypeptide chain topologies can be used in the multidomain sensors engineering [3]. Among them, there are both straightforward “tandem fusion” variant and more sophisticated schemes in which original FP and/or sensitive domain topology is artificially altered to provide better interdomain communication and consequently enhanced response characteristics. Regardless to the sensor topology and fluorescence output quantification principle (mostly ratiometric in Förster resonance energy transfer (FRET) sensors and intensiometric in single FP sensors), amino acid linkers are used to connect the sensor’s building blocks.

Functional role of linkers is dualistic. On one hand, they should act as the spacers providing correct folding and appropriate steric freedom of the sensor domains [4], on the other hand linkers take part in interdomain signal transmission either by limiting the space of domains conformational movements [5] or/and by transferring the mechanical force between the domains. Such a functional complexity alongside with the absence of a priori requirements to linker flexibility/rigidity and geometric configuration, as well as the difficulties in accurate prediction of the chimeric proteins structure and dynamics, makes linker design and optimization enormously challenging task.

Approaches to interdomain linkers optimization in FP-based sensors of diverse specificity have been well described in the literature, for instance [6,7,8,9,10,11,12,13,14,15,16]. Although there were attempts to develop common linker designing strategies suitable for the wide range of sensors [6,10], their modest outcomes cast doubts on the productivity of finding a universal solution in this area. We assume that the optimization of the linkers of a particular sensor is generally more promising. The new high-throughput techniques in sensors engineering and selection, where the libraries of chimeric proteins carrying linkers of different lengths and structures are generated and tested in automated mode [13,14,15,16,17], possess considerable potential remaining, however, of limited access to the fluorescent sensors developers.

Genetically encoded voltage indicators (GEVI) are the heterogeneous sensor family holding a special interest for the basic research in neurophysiology. Despite to a significant progress in GEVIs development [17,18,19,20], specifications of even top of these sensors remain suboptimal for the all-purpose routine use in *in vivo*-neurobiological studies (in contrast, for instance, to calcium [21] or dopamine [22] indicators). To date, there are totally around 10 GEVIs described as applicable for voltage detection in electrically excitable cells [17,18,19,20,23]. Most of them show the signal amplitude values associated with an action potential of 10% or less and has a practical temporal resolution limit at 100–200 Hz level. The fastest and the highest contrast GEVIs based on archaerhodopsin 3 [24] require exotic near-infrared lasers and very high fluorescence excitation intensities (0.1–1 kW/cm^2^) making the sensor imaging regime incompatible with the living brain tissue studies. To conclude, further work on GEVIs characteristics improvement is of importance.

The significance of interdomain linkers length and composition in GEVIs designing has been documented in a range of papers [9,18,25,26,27,28]. In particular, the development of the newest promising voltage sensors involved linker optimization [17,19,23].

Here we aimed to optimize amino acid linker in the recently described GEVI called VSD-FR189-188 [29]. In this construct, sensitive domain is represented by the voltage-sensing domain (VSD) from *Ciona intestinalis* voltage-sensing phosphatase (Ci-VSP) [30] proved itself as a good signal transducer and used extensively in the several GEVIs generations [26,31,32,33,34,35,36,37]. Reporter domain is based on the FusionRed, a low-phototoxic monomeric red fluorescent protein [38]. FusionRed C-terminal fragment (amino acids 189-233) is fused to Ci-VSP VSD N-terminus, and FusionRed N-terminal fragment (1-188)—to the VSD C-terminus, thus forming “insertion-into-circularly permuted FP” polypeptide chain topology. Being a relatively bright intensiometric sensor, emitting in the red part of spectrum and showing well-pronounced plasma membrane localization, VSD-FR189-188 would have become a probe of choice for the wide range of neuroscientists armed with a middle-level microscopic equipment, were it not of a relatively low contrast (ΔF/F~2% per 160 mV) and slow (tau~25 ms). Taking into account that, the “insertion-into-cpFP” topology itself can provide an efficient interdomain communication, we suggested that the optimization of interdomain polypeptide linker could be a key requirement for improvement of the sensor characteristics.

## 2. Materials and Methods

### 2.1. Genetic Engineering

The plasmids encoding the VSD-FR189-188 indicator with modified linkers were constructed on a basis of the original pVSD-FR189-188 [29].

To engineer sensor variants with 10, 17, 19 and 21 amino acid (aa) linker, the original 25 aa linker (EERIDIPEISGLWWGENEHGVDDGR) between the voltage-sensing domain and C-terminal fragment of FusionRed (189-233) was substituted by the synthetic oligonucleotides (sequences are shown in Supplementary Table S3) using KpnI/BamHI restriction endonuclease sites.

VSD-1,0,2,3,4,5 variants were engineered using overlap-extension polymerase chain reaction (PCR). FusionRed C-terminal fragment and voltage-sensing domain were PCR-amplified using primer pairs in which 5′-tail of FusionRed reverse primer had a sequence overlap with 5′-tail of the VSD forward primer. In case of VSD2,3,4,5 these 5′-primer regions contained a linker sequence. Mixed FusionRed 189-233 and VSD PCR-products were then melted/annealed, passed through 5 elongation cycles, and used as a PCR-template for amplification of a fused FusionRed 189-233-VSD fragment with a pair of terminal (FusionRed 189-233 forward and VSD reverse) primers (oligos and their sequences are listed in Supplementary Table S4). Final PCR products were subcloned into the plasmid containing FusionRed 1-188 fragment using NheI/SpeI restriction sites.

### 2.2. Maintenance and Transfection of HEK Cells

Human embryonic kidney HEK293 cell line [39] was from the Institute of Bioorganic Chemistry collection of cell lines.

HEK293 cells were used for both primary testing and additional electrophysiological experiments with GEVIs described here.

Cells were maintained in a humidified incubator at 37 °C with 5% CO_2_ and grown in complete Dulbecco’s modified Eagle’s medium DMEM medium (PanEco, Moscow, Russia) supplemented with 10% fetal bovine serum (Sigma, St. Louis, MO, USA), 4 mM L-glutamate, 10 U/mL penicillin and 100 mkg/mL streptomycin (PanEco, Moscow, Russia). The day before transfection, cells were seeded onto glass bottom dishes (Fluorodish, World Precision Instruments, Sarasota, FL, USA) and onto 8-mm-diameter coverglasses in 24-well plates, then grown to 70–80% confluence.

Transfection was performed using FuGene 6 (Promega, Madison, WI, USA) for primary testing and with Lipofectamine 2000 (ThermoFisher, Waltham, MA, USA) transfection reagents for neurophysiological recordings according to the manufacturer’s manuals. Fluorescent visualization was performed 48 h after transfection.

### 2.3. Primary Testing

All variants were expressed in HEK293T cells. Fluorescence checks for each linker variant were performed 48-h post-transfection. We monitored the fluorescent signal level and correct localization of the probe (it is important not to affect membrane localization during sensor optimization).

### 2.4. Electrophysiological Recording

Optical imaging and electrophysiological recording of HEK293 cells were performed at 48 h post-transfection in a perfusion chamber with the bath temperature kept at 22 ± 2 °C and perfused at a constant rate of 3 mL/min. A coverslip with HEK293 cells was placed in the recording chamber of an upright fluorescence microscope and superfused with bath solution (in mM: 125 NaCl, 25 NaHCO_3_, 27.5 glucose, 2.5 KCl, 1.25 NaH_2_PO_4_, 2 CaCl_2_ and 1.5 MgCl_2_, pH 7.4) pre-aerated with 95% O_2_, 5% CO_2_. Electrophysiological recordings were taken in voltage-clamp mode. Borosilicate glass electrodes (resistance of 5 MΩ) were filled with a solution containing (in mM) 132 K-Gluconate, 20 KCl, 4 Mg-ATP, 0.3 Na_2_GTP, 10 Na-Phosphocreatine, 10 HEPES, pH 7.25 (all from Sigma, St. Louis, MO, USA). Voltage steps were applied in whole-cell voltage clamp mode with npi ELC-03XS amplifier driven with a DigiData 1440A ADC board (Molecular Devices, San Jose, CA, USA). For precise positioning of the patch pipette, the rig was equipped with a motorized micromanipulator (Luigs and Neumann, Ratingen, Germany) mounted on an air table.

### 2.5. Optical Imaging of Membrane Potential

The fluorescence was recorded at 300–1000 fps rate with a high speed charge-coupled device (CCD) camera (NeuroCCD 80 × 80 or NeuroCCD-SM256 256 × 256, RedShirtImaging LLC, Decatur, GA, USA) attached to an upright Olympus BX51WI microscope equipped with a water immersion fluorescent objective (LUMPlanFl 40x 0.8 NA or LUMPLFLN 60x 1.0 NA) and powered by a LED (UHP-T-545-SR-LN-DF, Prizmatix, Israel, central wavelength 545 nm) at a narrow band-pass (a dichroic mirror, 565 nm, and 605/55 emission filter). We used Neuroplex 7.2 software (RedShirtImaging) for data acquisition and programming of synchronizing transistor–transistor logic (TTL) signals. The measured optical signal reflected the change in fluorescence relative to its mean value (∆F/F).

### 2.6. Image Analysis and Statistics

To obtain optical traces, cell-shaped ROIs were selected to encompass each patched cell while minimizing the background region. We typically performed step-triggered temporal averaging of a small number (10–20) of trials to increase the S-to-N ratio for displaying purposes. The time courses were corrected for bleaching using linear regression [40,41]. We fitted the onsets of optical signals evoked by square pulses with single exponential functions using Clampfit 10 software (molecular devices). All data are presented as mean ± standard error of the mean (SEM).

## 3. Results

**Figure 1:**
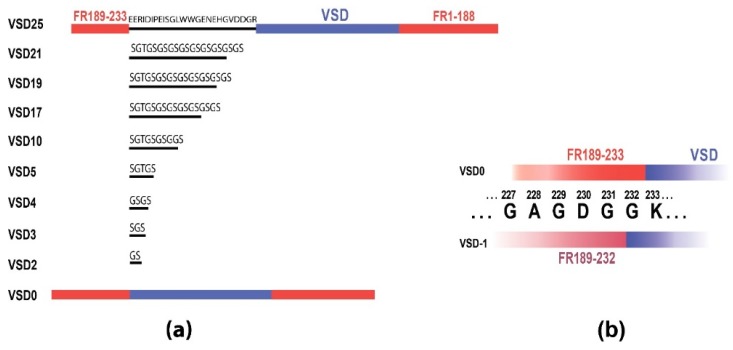
(**a**) Schematic representation of sensor variants construction with 10 different linker lengths, from original VSD25 to VSD0. FusionRed parts shown in red, VSD is blue and linkers are black. (**b**) The variant VSD-1 with the shortened FR189-232 part colored in pink.

In the original VSD-FR189-188 molecule, C-terminal FusionRed fragment (189-233) is connected to the Ci-VSP VSD through the linker, whereas N-terminal fragment (1-188) is cloned end-to-end (Figure 1). The original linker of 25 amino acids (aa) has a complex amino acid composition (EERIDIPEISGLWWGENEHGVDDGR) and is represented by a native sequence from Ci-VSP N-terminal domain. We supposed that in VSD-FR189-188 linker transmits mechanical force, produced by a voltage-induced VSD conformation shift, to the fluorescent protein molecule affecting its structure and leading to a fluorescence intensity change. Similar mechanism was earlier proposed for the VSFP3.1 single FP voltage indicator [42]. Thus, modest VSD-FR189-188 contrast and response speed could be attributed to the insufficient energy transduction capacity of the original 25 aa linker, and linker shortening would probably strengthen the interdomain interaction. To test this assumption, we decided to substitute 25 aa native linker with a set of shortened linkers built with the glycine-serine repeats ((GS)n) frequently used to connect structural parts in FP-based constructs. We thus replaced a complex sequence originating from the poorly annotated Ci-VSP N-terminus with a simple regular motif, which probably provides more transparent dependence between linker length and its conformation/flexibility.

**Figure 2:**
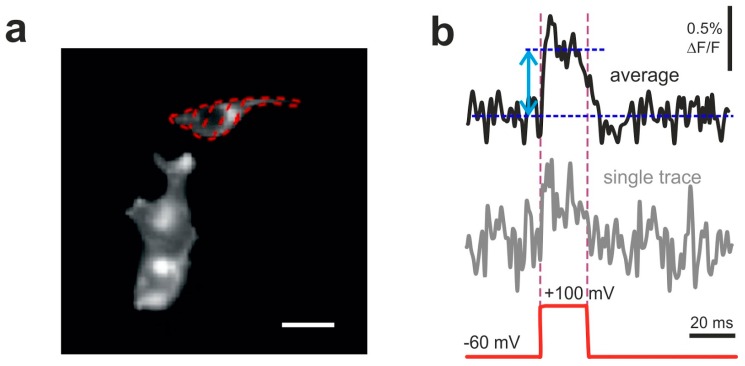
Examples of optical responses of HEK293T cells transfected with VSD2. (**a**) Dotted line encircles the analyzed region of interest (ROI). Scale bar: 30 um. (**b**) Optical traces (obtained from the ROI shown in a) of VSD2 signals evoked by a single voltage step of 160 mV amplitude (bottom red dashed line) repeated 10–20 times with 1 s intervals. Both single trial recording (bottom trace) and average of 10 traces (top bold trace) are shown. Double-headed arrow depicts the amplitude of averaged fluorescence signal measured against the baseline. Signal was recorded at 1 kHz framerate (1 ms exposure per frame).

Totally, we assembled nine sensor variants (Figure 1a) with the linker length ranging from 0 (no linker) to 21 amino acids (0, 2, 3, 4, 5, 10, 17, 19, 21; called VSD0-21 in the text), where VSD0 represents the variant with FusionRed 189-233 fragment cloned end-to-end with VSD. We also engineered VSD-1 variant (Figure 1b) that features VSD0 with FusionRed fragment reduced by one C-terminal amino acid (to 189-232). VSD0 and VSD-1 variants showed no fluorescence while expressed in mammalian cells revealing that the linker of at least 1-2 amino acids is necessary to provide correct FusionRed folding and/or chromophore maturation in the voltage-sensing constructs of this type. Remaining 8 constructs were expressed in HEK293 cells (Figure 2a, Supplementary Figure S2) where their fluorescence responses to the electric stimulation were measured in whole-cell voltage-clamp mode and compared to that of the original VSD-FR189-188 [29] (called VSD25 for the nomenclature uniformity).

To examine the signal amplitude and polarity in the set of probes mentioned above, we recorded optical signals of HEK293 cells evoked by the single voltage steps of 160 mV (Figure 2b, 10–20 single voltage steps with 1 s gaps were applied, averaged and analyzed) induced by current injection through the patch pipette in voltage clamp mode.

**Figure 3:**
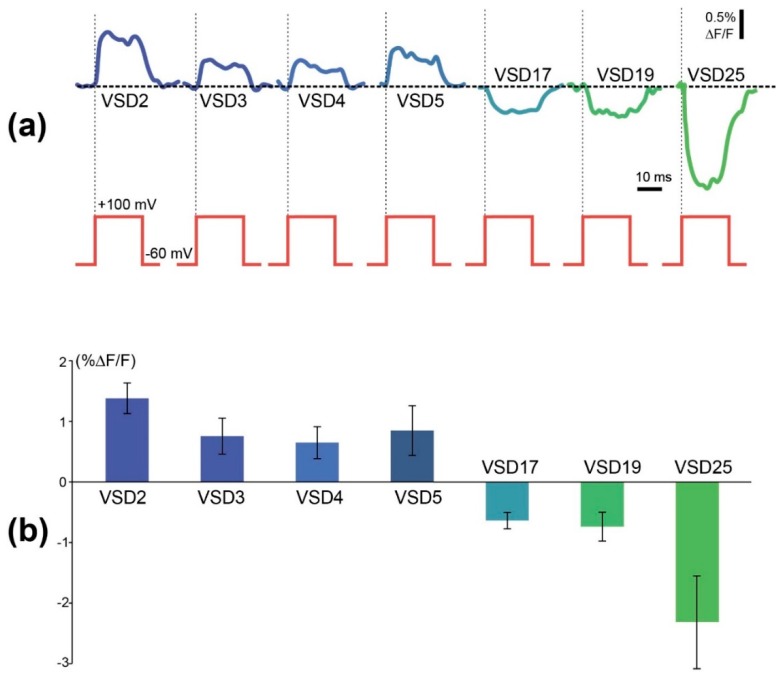
Comparison of amplitudes of optical responses of 7 different voltage-sensitive probes tested in similar conditions. (**a**) Examples of traces induced by voltage steps from −60 mV holding potential to +100 mV (upper red steps). Relative change in fluorescence of each probe was plotted below each test step of identical amplitude. Colors of optical traces correspond to the columns on histogram 3b and color-coded the tested probes. (**b**) Signal amplitudes of the probes measured as mean differences between signal amplitudes and baseline fluorescence of each probe (GEVI) are shown. Each column represents the average of 3–5 experiments +SEM.

In 6 out of 8 probes under examination, application of voltage steps resulted in step-like changes in a probe fluorescence, which differed in sign, amplitude, and a slope polarity (Figure 3, Supplementary Table S1). Since we didn’t detect the fluorescence response of VSD10 and VSD21, data for them are not shown.

These results demonstrate the complex influence of the linker length on the voltage sensor properties. In a strict sense, they contradict our initial hypothesis that linker shortening could cause a stronger FusionRed fluorescence shift, as the response contrast of the sensor variants with shortened linkers is even lower than in the original VSD-FR189-188 (aka VSD25). On the other hand, one can observe an easy-to-see trend in the response contrast/polarity dependence. Thus, VSD25 negative slope is changing to a positive one in between 17 and 5 amino acids. In a series of 5 to 2 amino acid linkers, there is a weak contrast increase. Taken together, these changes give a “cosine-like” dependence with the local negative maximum at 25 aa and the local positive maximum at 2 aa. Generally, the negative slope relationship between voltage change and fluorescence response is considered to be a disadvantage highly pervasive among the popular GEVIs (e.g., ArcLight, ASAP2f, and AceNeon) [43]. Earlier, response polarity “switch” was attained in Marina GEVI by a directed evolution of a fluorescent protein domain [43]. Here we found an alternative way to enable a positive slope ΔF/ΔV registration regime potentially applicable to a wide range of intensiometric FP-based GEVIs.

**Figure 4:**
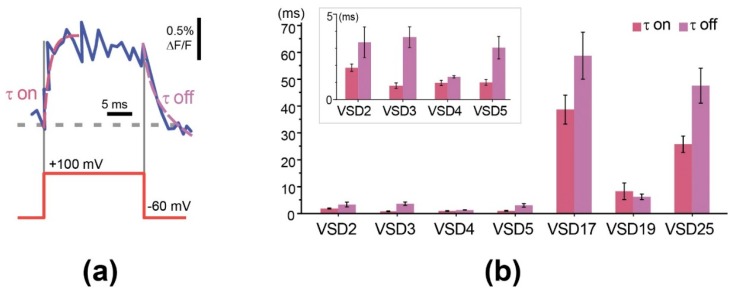
Characterization of VSD responsiveness by time constants. (**a**) Example of single exponential fitting (dashed magenta lines) of signal rise and decay at the beginning and end of 160 mV voltage step (red line). Optical response (noisy blue curve) was delayed from voltage command (red step below). The delay was characterized by tau parameters of single-exponential functions (dashed magenta line) fitted to the optical data. (**b**) Histograms show time constants (tau) ±SEM of the probes with different linker length obtained from single-exponential fits of signal onset (tau on) and decay (tau off), complete dataset is shown in Supplementary Table S2. Inset: time constants of VSD2-VSD5 at 5 ms scale.

We also evaluated the responsiveness of the probes (sensor response speed) using time constants obtained from single exponential fits of signal rise and decay at the beginning and end of a voltage step (Figure 4a).

Our experiments revealed that the constructs with linker length of 5 aa and shorter can reach submillisecond response speeds that are much (25–30-fold) faster than long-linker (VSD17, 19) variants and the parental VSD-FR189-188 responsiveness (~25 ms). Although we expected that linker shortening would rather increase the response contrast, responsiveness enhancement also falls into a paradigm of better interdomain communication. It is however hard to explain a jump-type time constant change from this point of view. Anyway, high response speed of GEVI is an important advantage necessary for in vivo applications, since live neurons in a natural context can fire action potential as fast as 600 Hz [44]. VSD2-VSD5 sensors showed responsiveness comparable to that of the best FP-based GEVIs–Mermaid [45], Ace2N-mNeon [18], Archon1 [17], VARNAM [19], and even of archaerhodopsin-based QuasAr2 [24]. With that, low response contrast of these constructs, insufficient for in vivo use, have stimulated further search for solutions.

VSD2—a variant with the highest contrast (~1.5%) among the high-speed short-linker constructs—has been chosen as a starting point for further optimization. Since we exhausted possibilities for further shortening of the linker between voltage-sensing domain and C-terminal FusionRed fragment, it was decided to truncate amino acids from C-terminus of the FusionRed N-terminal fragment (1-188). Particularly, 3 VSD2-based constructs with 2, 3, and 4 deleted FusionRed amino acids (called VSD2-186, VSD2-185, VSD2-184) were engineered (Supplementary Figures S1 and S2).

**Figure 5:**
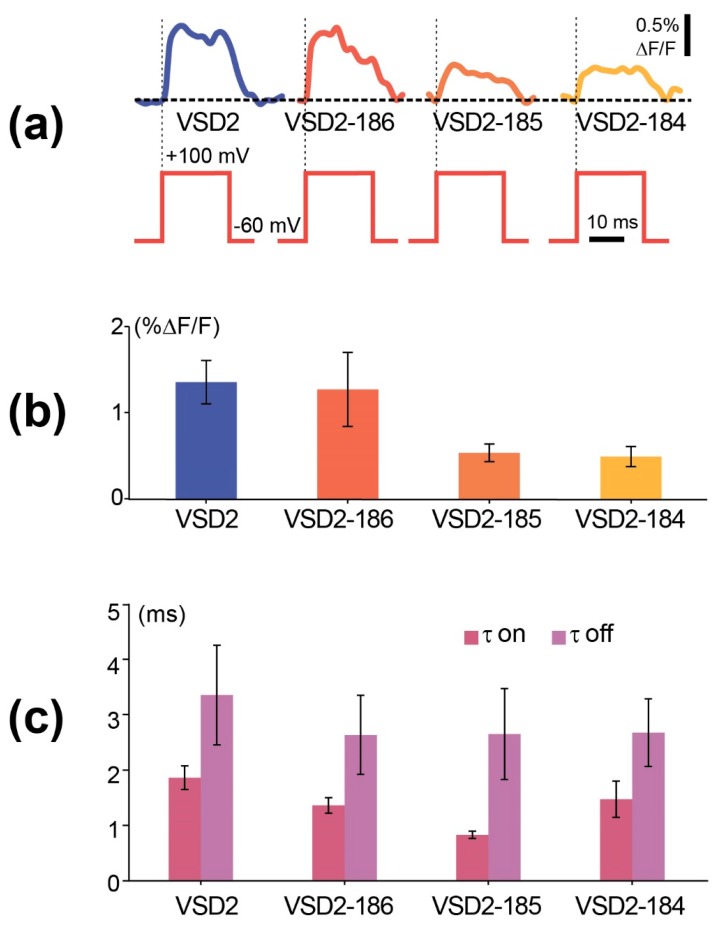
Functional characterization of GEVI variants in voltage-clamped HEK293 cells. (**a**) Fluorescence changes (bottom traces) to single voltage steps (top traces) recorded using four different VSDs whose linkers were modified from the C-termini (Supplementary Figure S1). (**b**) Signal amplitudes ±SEM measured against the baseline at the end of each voltage step. The colours of columns correspond to those of traces shown in (**a**). (**c**) Time constants (tau) ±SEM obtained from exponential fits of the ON-responses of the proteins.

We performed the standard HEK293 voltage-clamp experiments on the contrast and responsiveness evaluation with 2 aa linker and C-termini-truncated constructs and found that while their signal amplitudes either have not been significantly changed or even decreased relative to the parental VSD2 (Figure 5a,b, Supplementary Table S1), the time constant of VSD2-185 was substantially shorter than in VSD2 (0.83 vs. 1.86 ms, Figure 5c). Thus, FusionRed N-terminal fragment truncation did not affect fluorescence, albeit led to the relatively slight fluctuations of the sensor characteristics.

## 4. Discussion

Generally, VSD189-188 indicator linker shortening makes a significant impact to its voltage-sensing characteristics. Thus, this modification allows switching response polarity from impractical negative ΔV/ΔF slope to a convenient positive one, as well as increasing responsiveness 25–30-fold. On the other hand, while linker length influence on a signal amplitude is observable, it: (i) behaves in accordance with a complex nonlinear relationship that cannot be described by the simple speculative hypotheses on the more intense mechanical force interdomain transmission; (ii) is not expressed in a dramatic contrast increase. Accordingly, further efforts on the linker optimization could be directed, for instance, to a computer simulation of a structural behavior of the polypeptide fragments of different lengths and compositions using molecular dynamics and protein structure modelling methods. Probably such computer simulation-assisted linker analysis giving more information on the charge distribution and electrostatic interactions impact, chain rigidity, hydrophobicity, etc., would increase a chance for a successful implementation of the low-throughput approach to a voltage indicator designing.

Among the most straightforward experimental ways to evolve the results achieved here, we could suggest a study of linker composition impact in the chosen indicator constructs. Thus, behavior comparison of 25 aa glycine-serine linker construct with the original VSD25 would be of interest for revealing a role of the linker amino acid composition in determination of indicator properties. It looks also promising to perform an extensive mutational analysis of VSD2 linker. The fact that even local change in the sequences connecting GEVI functional domains can significantly influence its performance, was highlighted in the paper dedicated to Zahra 1 SE voltage indicator optimization where the authors have analyzed the impact of amino acid triad, formed from the restriction endonuclease site used for FP cloning, on the fluorescent signal [9]. Interestingly, the mentioned work also describes linker truncation analysis in Zahra 1 SE, which is built on the single FP and shares the same voltage-sensing core with our indicators. Their results to some extent contradict ours: thus, linker truncation led to increase in response contrast but did not affect responsiveness. The authors supposed that a shift in the construct voltage sensitivity was among the reasons for signal rise but the contradiction with our data also raises a question on the different ‘mechanics’ in the working cycle of the similar GEVIs carrying different fluorescent cores. Then, it is worth mentioning about a remarkable feature of the FusionRed tertiary structure, namely, a polypeptide chain break present in a large subpopulation of the mature protein [38]. Influence of this factor on the chromophore conformational flexibility and photobehavior of FusionRed-derived probes within GEVIs requires a special study. One can suppose that the FusionRed mutants lacking a chain cleavage being used in the voltage indicators would provide a signal dynamic range enhancement.

Finally, an interesting direction seems to us to analyze the GEVIs fluorescent signal in the lifetime domain instead of the spectral domain. Such an approach could allow quantitative analysis of the membrane voltage value, as has been done in several papers [20,46,47]. It is intriguing that changes in the fluorescent signal in spectral domain are not always strictly associated with changes in fluorescence lifetime [48], so the indicator with low contrast in the spectral domain is not necessarily poor in terms of the time domain.

## Supplementary Materials

Supplementary materials can be found at https://www.mdpi.com/1424-8220/19/13/2982/s1.

## Abbreviations

FP	Fluorescent protein
GEVI	Genetically encoded voltage indicator
FRET	Förster resonance energy transfer
Ci-VSP	*Ciona intestinalis* voltage-sensing phosphatase
VSD	Voltage-sensing domain
HEK293	Human embryonic kidney 293 cells
