# Supplementary material for: Red Fluorescent Genetically Encoded Voltage Indicators with Millisecond Responsiveness
Source: Sensors (Basel). 2019 Jul 6;19(13):2982. doi: 10.3390/s19132982 (PMC6651345; doi:10.3390/s19132982)
Supplement: Supplementary file 1 [file sensors-19-02982-s001.pdf]

Supplementary materials for:

Red fluorescent genetically encoded voltage indicators with millisecond responsiveness

Liubov A. Kost, Violetta O. Ivanova, Pavel M. Balaban, Konstantin A. Lukyanov, Evgeny S. Nikitin, Alexey M. Bogdanov

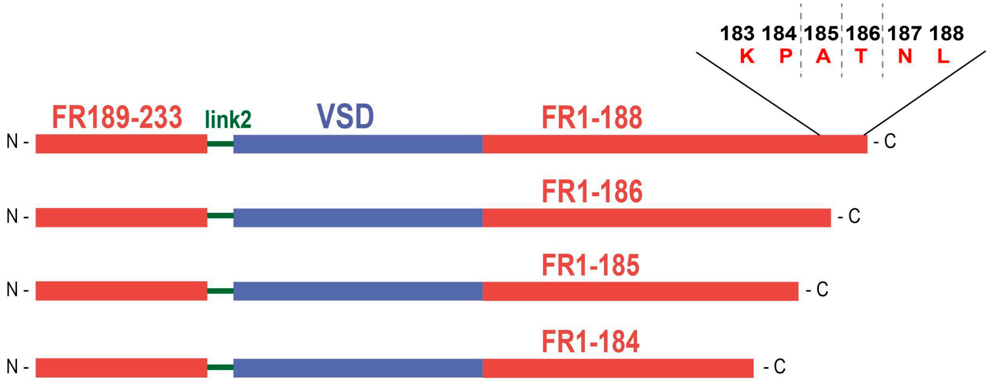

Supplementary Figure S1. VSD2 modifications - VSD2-186, VSD2-185, VSD2-184 - engineered by the cpFusionRed 1-188 C-terminus shortening. Red color shows two fragments of cpFusionRed, green represents interdomain linker part, voltage-sensing domain is shown in blue.

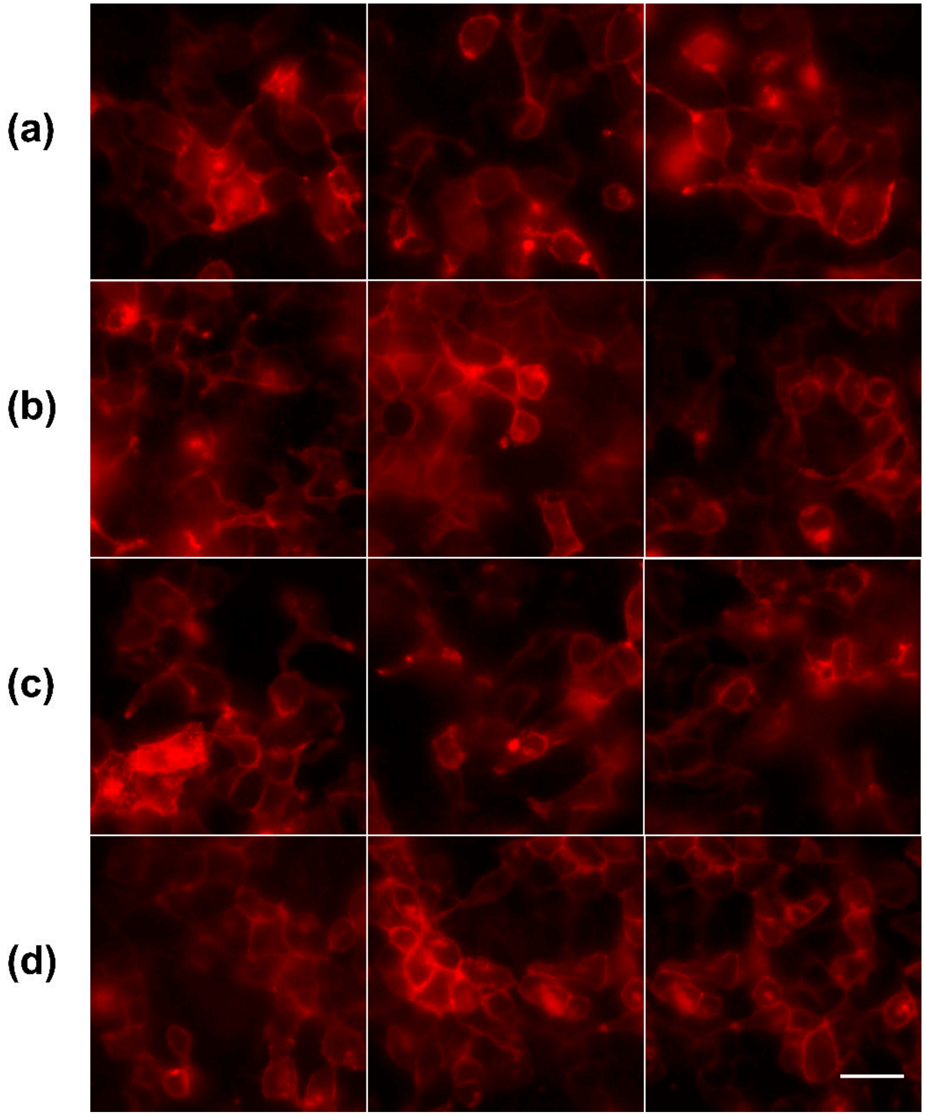

Supplementary Figure S2. Wide-field fluorescence images of live HEK293T cells expressing VSD-FR189-188-derived voltage indicators (a) VSD2, (b) VSD2-186, (c) VSD2-185, (d) VSD2-186. All the images were acquired 48 hours post transfection using BioRevo BZ-9000 (Keyence) epifluorescence microscope (Nikon CFI Plan APO VC 60x/ 1.40 Oil ∞/0.17 Dic N2 WD 0.13 Microscope Objective, RFP filter set TxRed. EX 560/40; DM 595; BA 630/60). Scale bar: 50 um.

Supplementary Table S1. Voltage probes response amplitudes analyzed by patch-clamp experiments in HEK293T cells. Signal amplitudes for each registered cell (ΔF/F, %) are shown. “No fluorescence” - transfection of the probe revealed no visible fluorescence; “no response” - the probes did not respond to the voltage steps.

Cell #	VSD -1	VSD 0	VSD 2	VSD 2-186	VSD 2-185	VSD 2-184	VSD 3	VSD 4	VSD 5	VSD 10	VSD 17	VSD 19	VSD 21	VSD 25
1	no fluorescence	no fluorescence	0.91	1.25	0.71	0.35	0.36	0.3	1.49	no response	-0.67	-0.49	no response	-2
2			1.37	1.10	0.36	0.48	0.4	0.55	0.41		-0.44	-0.91		-2.13
3			1.53	0.57	0.55	0.32	1	0.71	0.54		-0.63	-0.61		-1.13
4			1.19	1.32	0.39	0.7	0.99	0.8	0.65					-3.09
5			1.28	1.65										-2.17
Average			1.25	1.17	0.50	0.46	0.68	0.59	0.95		-0.58	-0.67		-2.10
S.E.M.			0.06	0.12	0.01	0.02	0.08	0.02	0.23		0.01	0.03		0.38

ΔF/F,%

Supplementary Table S2. Voltage probes responsivness analyzed by patch-clamp experiments in HEK293T cells. Tau on and tau off values for each registered cell (ms) are shown.

Cell #	VSD 2		VSD 2-186		VSD 2-185		VSD 2-184		VSD 3		VSD 4		VSD 5		VSD 17		VSD 19		VSD 25	
	ON	OFF	ON	OFF	ON	OFF	ON	OFF	ON	OFF	ON	OFF	ON	OFF	ON	OFF	ON	OFF	ON	OFF
1	1.62	6.14	1.06	1.12	1.02	4.96	1.11	1.46	1.15	2.33	0.93	1.47	0.67	2.69	29.70	76.12	13.40	4.90	18	68.26
2	1.38	4.62	1.82	4.20	0.79	1.72	2.11	4.33	1.03	3.32	1.38	1.32	0.90	2.58	38.00	51.79	8.80	8.25	26	39.87
3	1.57	2.45	1.42	1.30	0.71	1.30	0.74	2.70	0.56	3.68	0.96	1.15	0.99	1.94	48.40	48.36	2.70	5.51	20	34.13
4	2.45	2.48	1.08	2.10	0.80	2.61	1.94	2.21	0.49	5.28	0.63	1.37	1.45	4.94					32	57.30
5	2.30	1.08	1.44	4.46															33	38.45
Average	1.86	3.35	1.36	2.64	0.83	2.65	1.48	2.68	0.81	3.65	0.98	1.33	1.00	3.04	38.70	58.76	8.30	6.22	25.80	47.60
S.E.M.	0.21	0.90	0.14	0.71	0.06	0.82	0.33	0.61	0.17	0.61	0.15	0.07	0.16	0.66	5.41	8.74	3.10	1.03	3.04	6.50

Tau, ms

Supplementary Table S3. List of oligonucleotides used for engineering constructs with linkers 10, 17, 19 and 21 in this study. Pairs of sense and antisense oligonucleotides were melted and annealed to obtain double-stranded DNA fragments with the single-strand overhangs ready for ligation.

Name	Oligonucleotide sequence
10 KpnI GlySer5 BamHI sense	CGGCAGCGGCAGCGGCG
10 KpnI GlySer5 BamHI antisense	GATCCGCCGCTGCCGCTGCCGGTAC
15 KpnI GlySer10 BamHI sense	CGGCAGCGGCAGCGGCAGCGGCAGCGGCAGCG
15 KpnI GlySer10 BamHI antisense	GATCCGCTGCCGCTGCCGCTGCCGCTGCCGCTGCCGGTAC
20 KpnI GlySer15 BamHI sense	CGGCAGCGGCAGCGGCAGCGGCAGCGGCAGCGGCAGCGGCAGCGGCG
20 KpnI GlySer15 BamHI antisense	GATCCGCCGCTGCCGCTGCCGCTGCCGCTGCCGCTGCCGCTGCCGCTGCCGGTAC
Olig19 Sense	CGGTTCTGGCAGTGGATCGGGTTCGGGCTCAGGGTCTGGTTCAG
Olig19 Antisense	GTACCGGTTCTGGCAGTGGATCGGGTTCGGGCTCAGGGTCTGGTTCAGGATC
Olig21 Sense	CGGCAGCGGTTCTGGCAGTGGATCGGGTTCGGGCTCAGGGTCTGGTTCAG
Olig21 Antisense	GTACCGGCAGCGGTTCTGGCAGTGGATCGGGTTCGGGCTCAGGGTCTGGTTCAGGATC

Supplementary Table S4. PCR primers used for engineering VSD2, 3, 4, 0 and -1 variants.

Name	Oligonucleotide primers sequence
FR189 Nhe for	AAAAGCTAGCCGCCACCATGAAGATGCCCGG
VSD Spe rev	AAAAACTAGTCTGTGATATTGTTCTTCTGCT
VSD overlap for	ATGGAGATACCTACTACTGGTG
FR232 overlap rev	CACCAGTAGTAGGTATCTCCATACCTCCATCACCAGCGC
FR233 overlap rev	GCGCTGGTGATGGAGGTAAAATGGAGATACCTACTACTGGTG
Link 2 BamH rev	GTGGCGCTGGTGATGGAGGTAAAGGATCC
Link 3 S BamH rev	GCGCTGGTGATGGAGGTAAAAGUGGATCC
Link 4 GS BamH rev	GCGCTGGTGATGGAGGTAAAGGUAGUGGATCC
